# A questionnaire-based (UM-PDHQ) study of hallucinations in Parkinson's disease

**DOI:** 10.1186/1471-2377-8-21

**Published:** 2008-06-20

**Authors:** Spiridon Papapetropoulos, Heather Katzen, Anette Schrag, Carlos Singer, Blake K Scanlon, Daniel Nation, Alexandra Guevara, Bonnie Levin

**Affiliations:** 1 Divisions of Movement Disorders Department of Neurology, University of Miami, Miller School of Medicine Miami, FL, USA; 2 Division of Neuropsychology Department of Neurology, University of Miami, Miller School of Medicine Miami, FL, USA; 3 Department of Clinical Neurosciences, Royal Free and University College Medical School, University College London, London, UK

## Abstract

**Background:**

Hallucinations occur in 20–40% of PD patients and have been associated with unfavorable clinical outcomes (i.e., nursing home placement, increased mortality). Hallucinations, like other non-motor features of PD, are not well recognized in routine primary/secondary clinical practice. So far, there has been no instrument for uniform characterization of hallucinations in PD. To this end, we developed the University of Miami Parkinson's disease Hallucinations Questionnaire (UM-PDHQ) that allows comprehensive assessment of hallucinations in clinical or research settings.

**Methods:**

The UM-PDHQ is composed of 6 quantitative and 14 qualitative items. For our study PD patients of all ages and in all stages of the disease were recruited over an 18-month period. The UPDRS, MMSE, and Beck Depression and Anxiety Inventories were used for comparisons.

**Results and Discussion:**

Seventy consecutive PD patients were included in the analyses. Thirty-one (44.3%) were classified as hallucinators and 39 as non-hallucinators. No significant group differences were observed in terms of demographics, disease characteristics, stage, education, depressive/anxiety scores or cognitive functioning (MMSE) between hallucinators and non-hallucinators. Single mode hallucinations were reported in 20/31 (visual/14, auditory/4, olfactory/2) whereas multiple modalities were reported in 11/31 patients. The most common hallucinatory experience was a whole person followed by small animals, insects and reptiles.

**Conclusion:**

Using the UM-PDHQ, we were able to define the key characteristics of hallucinations in PD in our cohort. Future directions include the validation of the quantitative part of the questionnaire than will serve as a rating scale for severity of hallucinations.

## Background

Hallucinations occur in 20–40% of Parkinson's disease (PD) patients receiving symptomatic therapy [1]. Although potentially treatable by anti-parkinsonian drug adjustments and the use of atypical antipsychotics [2], hallucinations have been associated with unfavorable clinical outcomes such as nursing home placement and increased mortality [3,4].

Hallucinations, like other non-motor features of PD, are not well recognized in routine clinical practice, either in primary or in secondary care, and are frequently missed during consultations [5]. Standardized PD rating scales such as the Unified Parkinson's disease Rating Scale (UPDRS) part I [6] have low sensitivity to detect hallucinations and other psychotic symptoms[7]. Symptom and disease specific instruments such as the Parkinson's Psychosis Rating Scale (PPRS), [8] the non-motor symptom questionnaire (NMSQuest) [5] or the Parkinson's Disease-Psychosocial questionnaire (SCOPA-PS) [9] assess hallucinations only in a few sub-items. Other PD specific instruments that may offer a more detailed characterization of hallucinations, such as the Rush Parkinson's Disease Behavioral Interview [10] are not easily administered in the busy clinical settings and generic rating scales/questionnaires that were designed to address organic brain psychosis and/or neuropsychiatric manifestations (ie the Neuropsychiatric Inventory [11]) are of limited use in characterizing PD-associated hallucinations.

To date there is no instrument that allows for a focused, comprehensive assessment of the characteristics of hallucinations in PD for the clinician or for use as a research outcome measure. We therefore developed the University of Miami Parkinson's disease Hallucinations Questionnaire (UM-PDHQ), a 20-item questionnaire to be used as a screening instrument to assess hallucinations in PD. This pilot study, part of an initiative begun at the University of Miami, Miller School of Medicine sought to quantify the type and presence of hallucinations in a clinic population while controlling for disease factors, depression, anxiety and medication.

## Methods

### The University of Miami Parkinson's disease Hallucinations Questionnaire (UM-PDHQ)

The UM-PDHQ is a 20-item clinician-administered questionnaire that is completed during a structured interview (see Additional file 1). The 20 items were derived through consultations with PD patients, caregivers, and a panel of experts including 4 movement disorders specialists, 1 geriatric psychiatrist, 3 neuropsychologists, 1 nurse specialist and 1 neuro-opthalmologist. The core group met on a monthly basis for a period of 6 months to produce a working questionnaire and subsequent revisions were made to improve ease of administration. Questions were divided into two groups; a quantitative group that consists of 6 questions (modality, frequency, duration, insight, emotional burden) and a qualitative group that consists of 14 questions. The first item is a gating question to assess the presence or absence of hallucinations. It is derived from modifications to item 3 of the UPDRS part I, and item 14 of the non-motor symptom questionnaire (NMSQuest) for PD [5]. The quantitative section of the UM-PDHQ is currently being validated and is used only for descriptive purposes as part of this study. The complete questionnaire is presented in the Appendix.

### Patients

For the purposes of our pilot study, PD patients of all ages and in all stages of the disease were recruited. PD patients diagnosed using the U.K. PD Brain Bank criteria [12] were evaluated at the Department of Neurology, University of Miami, Miller School of Medicine. A movement disorders specialist evaluated degree of motor impairment by means of the Unified Parkinson's disease rating scale (UPDRS) [13], side of symptom onset, and a modified Hoehn and Yahr rating [13] as part of a comprehensive neurological examination. In patients with "on-offs", "on" UPDRS scores were used in the analysis. Items derived from the activities of daily living (ADL) (UPDRS part II) and motor features (UPDRS part III) subscales were used to determine PD subtype, according to the method proposed by Jankovic and colleagues [14]. The subtypes are referred to as tremor-dominant PD (TD) and postural instability/gait-dominant (PIGD). No patients had evidence of hallucinations as part of a drug-induced toxic delirium or complication of a superimposed medical illness. All patients were screened for mental function using the Mini Mental State Examination (MMSE) [15]. Depressive symptomatology was assessed during the clinical interview with a neuropsychologist and was objectively measured with the Beck Depression Inventory – Second Edition (BDI-II), a 21-item self-report questionnaire of depressive symptoms [16]. The BDI has been validated for use in PD [17,18] and compares favorably with other depression screening measures [19]. Patients were evaluated for anxiety using the Beck Anxiety Inventory (BAI) [20]. The UM-PDHQ, BDI and BAI were administered by a trained clinical neuropsychologists blinded to the results of the neurological examination and UPDRS. Our local institutional review board approved this study.

### Statistical analyses

Statistical analysis was performed using the SPSS for Windows release 11.0 (SPSS Inc, Chicago, Ill., USA). Comparisons of continuous data were carried out using Mann-Whitney U test for 2 samples or Kruskal-Wallis tests for more samples. Categorical data were analyzed using Chi-square with Yates corrected p-value or Fisher exact 2-tail p value. All significance tests were two-tailed and were conducted at p < 0.05.

## Results

### Participant characteristics

Seventy consecutive PD patients (46 men and 24 women) seen in our center were included in the analyses. Mean age at study inclusion was 64.3 ± 10.2 years and mean age of PD onset was 55.1 ± 12.1 years. Thirty seven (52.9%) patients had right-side PD onset. Mean Hoehn and Yahr disease stage was 2.5 ± 0.7, mean MMSE was 25.6 ± 4.5, mean BDI-II total score was 16.3 ± 12.7 and mean BAI score was 18.7 ± 11.5

Using the UM-PDHQ 31 (44.3%) patients were classified as hallucinators and 39 were classified as non-hallucinators. The UPDRS part I identified 26 (37.1%) and fail to report hallucinations in 5 patients. Significant group differences were not observed in terms of gender, age at examination, age at PD onset, side of PD onset, PD duration, disease stage, education, depressive/anxiety scores or overall cognitive functioning (MMSE) between hallucinators and non-hallucinators (Table 1). However, UPDRS total, part I, II and III scores were significantly higher in hallucinators (Table 1). There were no differences in disease subtypes (TD vs PIGD) between hallucinators and non-hallucinators (p = 0.1). There were no differences between groups in use of antiparkinsonian medication (L-dopa dose, DA, MAO-B inhibitors, anticholinergics, COMT inhibitors) (Table 2). Five (16.1%) patients with hallucinations were being treated with antipsychotics, whereas none of the patients without hallucinations (0%) were on antipsychotics (p = 0.01). The use of antidepressants was similar between groups. Comparisons between active and past hallucinators did not reveal statistical significant results in any of the demographic and clinical parameters tested.

**Table 1:** Demographics and phenotypic features of our patient population

Variable	All patients	Non Hallucinators N = 39	Hallucinators N = 31
Gender (%male)	46 (65.7%)	25 (64.1%)	21 (67.7%)
Age at onset in yrs (SD)	55.1 (12.1)	55.8 (11.8)	54.4 (12.5)
Age at exam in yrs (SD)	64.3 (10.2)	64.3 (10.5)	63.9 (10.0)
Range	39–84	44–83	39–84
Disease Duration in years (SD)	9.0 (5.4)	8.5 (5.3)	9.5 (5.6)
Hoehn and Yahr stage (SD)	2.5 (0.7)	2.4 (0.7)	2.5 (0.7)
(min-max)	1–5	1–5	1.5–4
Hoehn and Yahr 1 (%)	4 (5.7)	4 (10.3)	0
Hoehn and Yahr 1.5 (%)	5 (7.1)	3 (7.7)	2 (6.5)
Hoehn and Yahr 2 (%)	21 (30.0)	12 (30.8)	9 (29)
Hoehn and Yahr 2.5 (%)	23 (32.9)	12 (30.8)	11 (35.5)
Hoehn and Yahr 3 (%)	8 (11.4)	4 (10.3)	4 (12.9)
Hoehn and Yahr 4 (%)	8 (11.4)	3 (7.7)	5 (16.1)
Hoehn and Yahr 5 (%)	1 (1.4)	1 (2.6)	0
UPDRSI*	4.2 (2.7)	3.5 (2.3)	5.3 (2.9)
UPDRSII*	13.6 (7.5)	11.2 (5.9)	17.2 (8.2)
UPDRSIII*	22.1 (10.0)	19.1 (10.3)	26.1 (8.1)

Side of onset			
Right	37 (52.9%)	21 (53.8%)	16 (51.6%)
Left	27 (38.6%)	16 (41.0%)	11 (35.5%)
Bilateral	5 (71.%)	2 (5.1%)	3 (9.7%)
Unknown	1 (1.4%)	0	1 (3.2%)

Education in yrs (SD)	13.0 (4.4)	12.8 (4.2)	13.2 (4.9)
MMSE score (SD)	25.6 (4.5)	26.1 (4.2)	25.0 (4.7)
(min-max)	10–30	14–30	10–30
BDI (SD)	16.3 (12.7)	15.1 (11.1)	17.9 (14.7)
BAI (SD)	18.7 (11.5)	16.5 (10.2)	21.5 (12.6)

*Statistically significant (p > 0.05)

Yrs: years, SD: Standard deviation, UPDRS: Unified Parkinson's disease rating scale, MMSE: Mini Mental State Exam, BDI: Beck's Depression Inventory, BAI: Beck's Anxiety Inventory

**Table 2:** Medication profile

Group	Non Hallucinators	Hallucinators
	N = 39	N = 31
L-dopa	38 (97.4%)	30 (96.8%)
Dopamine Agonist	25 (64.1%)	18 (58.1%)
Amantadine	9 (23.1%)	7 (22.6%)
Anticholinergics	4 (10.3%)	4 (12.9%)
COMT inhibitors	7 (17.9%)	4 (12.9%)
MAO-B inhibitors	2 (5.1%)	3 (9.7%)
Cholinesterase inhibitors	4 (10.3%)	1 (3.2%)
Antipsychotics*	0	5 (16.1%)
Antidepressants	12 (30.8%)	11 (35.5%)

*p = 0.01

### Characteristics of hallucinators

**Figure 1:**
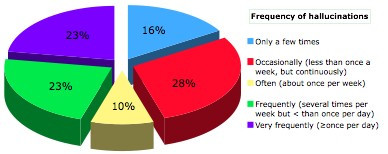
Overall frequency of hallucinations.

**Figure 2:**
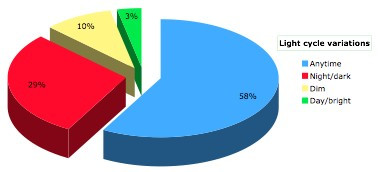
Frequency of hallucinations according to light cycle.

Of the 31 patients more than half (56%) experienced hallucinations once per week or more. The reported frequency of hallucinations is presented in Figure 1. Hallucinations were instantaneous (<1 sec) in 10 (32.3%), of medium duration (<10 sec) in 18 (58.1%) and of prolonged duration (>10 sec) in 1 PD patient. Two patients could not specify the duration of their experiences. None of the hallucinators reported current eye disease other than corrected refractory problems. Two out of 31 (6.5%) patients reported that their hallucinations resulted after alterations in their treatment. In patients experiencing on-off motor fluctuations, hallucinations were present more often during the on phase (13 during on and 2 during off). Fifteen hallucinators (48.4%) were classified in the TD group and 16 (51.6%) in the PIGD group. Onset of hallucinations was sudden in the majority of patients (21/31). Only 4 reported gradual onset and the rest were not sure of onset type. Hallucinations were less frequently associated with the light cycle (Figure 2).

When patients were questioned as to whether they thought their hallucinations were real (non-retained insight), 11 (35.5%) patients responded "no", 7 (25.5%) thought that they were sometimes real, 10 (32.3%) responded "yes" and the rest could not tell. Surprisingly, there were no differences (p = 0.99) in the mean MMSE scores between these groups (24.2 ± 5.2 vs 25.2 ± 5.0 vs 24.9 ± 4.7). None of the other parameters were significantly different. In 13 (42%) patients hallucinations were described as friendly/indifferent but in 18 (58%) some measure of distress was reported (mild in 19.4%, moderate in 16% and severe in 22.6%). Patients distressed by hallucinations were more frequently (p = 0.02) on antidepressants (no distress: 2; 18.2% vs distress 9; 81.8%). None of the other parameters tested reached significance although BDI-II and BAI scores were higher in patients admitting distress due to hallucinations (BDI-II 21.6 ± 15.9 and BAI 25.2 ± 15.7) versus patients without distress (BDI-II 14.0 ± 12.7 and BAI 17.8 ± 7.6). Only 12/31 (38.7%) patients admitted that sensations experienced during hallucinations were familiar.

**Figure 3:**
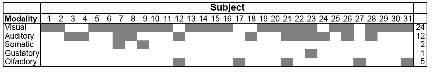
**Hallucinatory experiences/patient.** Columns represent individual patients and rows represent different hallucinatory modalities.

Single mode hallucinations were reported in 20/31 (visual 14, auditory 4, olfactory 2) whereas multiple modalities were reported in 11/31 patients. Frequencies of different modalities are presented in Figures 3. Five (16.1%) patients admitted that their visual experiences were accompanied by sound, usually voices. Comparisons between patients with hallucinations in one versus multiple modalities did not reveal statistical significant results in any of the parameters tested. Seven patients (22.5%) with hallucinations did not report a visual component. All patients with non-visual hallucinations were male versus 14/24 (58.3%) in the visual hallucinations group (p = 0.04). Other comparisons between visual and non-visual hallucinators did not reveal significant results in the parameters tested.

**Figure 4:**
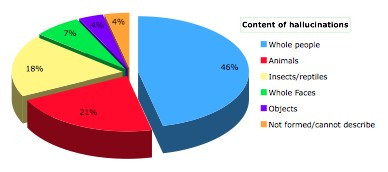
Content of hallucinations.

Content of visual hallucinations is presented in Figure 4. The most common hallucinatory experience was a whole person followed by small animals, insects and reptiles. Visual hallucinations were usually of normal size with only 3 patients reporting size distortion. Eleven out of 24 patients (46%) experiencing visual hallucinations reported that their hallucinations were considered "achromatic". Only 2 reported transparent images. A selected description of hallucinatory experiences is presented in Table 3.

**Table 3:** Descriptions of hallucinatory experiences in Parkinson's disease

"I smell cleaning products"
"I see fragmented bodies"
"I hear conversations; usually people arguing"
"I hears voices"
"I see unfamiliar male faces; I also see little bears"
"I hear my parents' voices and "psst psst psst" sounds that remind me of a bird"
"I see people running, human silhouettes, dogs that make noises. I also hear voices"
"I occasionally think the telephone is ringing when it is not"
"I see a shadow and feel like someone is sitting on chest"
"I see my neighbor's children"
"I see shadows of people"
"I smell medicine"
"I see spider webs and a luminous ball"
"I smell gas"

## Discussion

Hallucinations in PD have received growing attention since the introduction of chronic treatments of dopamine restoration which increased their frequency from 5% in the pre-L-dopa era [21-23] to around 40% in chronically treated patients at least 3 years into their disease [1]. Hallucinations may be considered a characteristic of α-synucleinopathies, a group of diseases associated with excessive accumulation of α-synuclein in the CNS that includes PD, dementia with Lewy bodies and multiple system atrophy [24,25] and a red flag for Lewy body pathology [26].

Although there is an extensive literature in this area, lack of consistency and standardization of assessment approaches has significantly biased comparison data across studies and made it difficult to conclude about prevalence rates, content, classification, risk factors and treatment outcomes [1]. Only recently a set of diagnostic criteria for psychotic symptoms that include hallucinations in PD, were proposed by a panel of NIH-sponsored experts. This working group highlighted the lack of relevant instruments/scales [27]. Few studies i.e. [28,29] have used validated scales or structured interviews, including the UPDRS I thought disorder subscale score [6] and the Rush Parkinson's Disease Behavioral Interview [10] respectively. Our study confirmed previous findings that showed that the UPDRS thought disorders subscale is not considered the most sensitive instrument to detect psychotic symptoms in PD [7]. The Rush Behavioral interview primarily assesses the severity of hallucinations of all modalities focusing on frequency in the past month: severity score: 0 = none, 1 = less than once weekly, 2 = once or twice weekly, 3 = at least three times weekly and does not ascertain detailed information about the type and nature of hallucinations. Severe hallucinations are defined as a score of 3. To this end we developed the UM-PDHQ to systematically study hallucinatory experiences in PD in an outpatient clinic setting.

The UM-PDHQ confirmed previous findings on the prevalence of hallucinations; the prevalence in our cohort (44.3%) was similar to other clinic-based prospective studies and somewhat higher than previously reported in community studies [30,31]. Studies focusing on a single hallucinatory modality, mainly visual [32,33], have consistently reported lower prevalence of hallucinations as they fail to consider auditory, olfactory, somatic and gustatory modalities. Among hallucinators, the cumulative percentage of reported non-visual modalities was 54.8%.

In PD, hallucinatory experiences tend to be present intermittently, lasting seconds to minutes at a time [34]. Their frequency can vary, but most studies register the presence of hallucinations when they occur at least one time per week. Often, they occur several times per day [27]. The typical hallucinatory experience in our clinic-based cohort was that of a non-threatening, familiar or unfamiliar solid, real sized, colored or colorless human body that appeared suddenly anytime during the day and disappeared a few seconds later. Little animals and insects were also common. A variety of other non-typical hallucinatory sensations were very frequently described. Therefore one should not consider hallucinatory experiences in PD uniform. Simplistic classifications such as benign-malignant, simple-complex or minor-major should be avoided until a scaling method has been developed and validated. We also found that hallucinations were unrelated to light cycle in the majority of patients. Previous reports have suggested that hallucinations tend to occur during times of low ambient stimulation, most typically in the evening or when the patient is alone in a quiet environment [27].

Hallucinations lacking a visual component were reported in 7 out of 70 patients with an overall prevalence of 10%. Several other systematic prospective studies have pointed out the increased frequency of non-visual hallucinatory modalities in PD [35,36]. Their content may vary considerably [37,38]. Non-visual hallucinations may often consist of whispers or music, as opposed to the threatening voices reported in schizophrenia; however, some cases of threatening auditory hallucinations have been reported in PD [39]. Tactile, olfactory and gustatory hallucinations have also been reported; however less commonly, and they tend to co-occur with visual hallucinations [40,41].

There were no differences detected between hallucinators and non-hallucinators in demographic and neuropsychiatric profiles. Confirming previous observations from our group, hallucinations interfered with activities of daily living and were related to more severe symptomatology as measured by the UPDRS [32]. As expected, hallucinators were more frequently prescribed antipsychotics. Although cognitive impairment and dementia have been frequently reported as risk factors for hallucinations in PD [31] no such association (as measured by the MMSE score) was detected in our study. Our results are in line with those of another interview-based prospective study that found no association between MMSE scores and hallucinations at the conclusion of a 4-year study [29]. In our cohort loss of insight regarding hallucinatory experiences was unrelated to cognitive status. Loss of insight has been associated with the "continuum hypothesis" that suggests a progression of psychotic symptoms from sleep disturbances to hallucinatory experiences to full-blow psychosis [42]. However, our findings on insight and spontaneous remission of hallucinations in 9/31 (29%) of patients coupled with lack of a REM behavior sleep disorder in some hallucinators [43,44] suggest that this theory may not apply for all patients. In using only the MMSE as our measure of cognitive status, we did not examine particular cognitive domains that may have differentiated hallucinators from non-hallucinators. BDI scores and BAI scores were not significantly different between hallucinators and non-hallucinators. It is unclear from our data whether the higher prevalence of antidepressant use among hallucinators who were distressed by their hallucinations as compared to those not distressed was either a contributor to or a cause of the distress.

Our study has certain limitations; the UM-PDHQ in its current form does not provide an overall score of severity and is not a graded or rating instrument. Instead, it is a screening tool designed to draw attention to the presence of hallucinations and initiate further investigation. In our study cognitive status was assessed using the MMSE which is a brief screening measure that is known to be less sensitive to subcortical functions [45]. However, examining the relationship between cognitive decline and hallucinations was beyond the scope of this pilot study. We are currently collecting more detailed neuropsyhological data in a larger cohort of patients to examine this important question.

## Conclusion

Using the UM-PDHQ during a structured interview we were able to define in depth the key characteristics of hallucinations in a pilot cohort of PD patients. Future directions include the validation of the questionnaire and the development of a disease and symptom rating scale for severity of hallucinations. The UM-PDHQ may be modified in the future as additional data become available. Valid criteria for psychotic symptoms in PD combined with disease specific questionnaires and scales may provide the necessary endpoints that meet regulatory needs for future clinical trials on the subject.

## Abbreviations

Unified Parkinson's disease Rating Scale: UPDRS; Parkinson's Psychosis Rating Scale: PPRS; Non-motor symptom questionnaire: NMSQuest; Parkinson's Disease-Psychosocial questionnaire: SCOPA-PS; University of Miami Parkinson's disease Hallucinations Questionnaire: UM-PDHQ; Tremor-dominant PD: TD and Postural Instability/Gait-Dominant (PIGD); Activities of Daily Living: ADL; Beck Anxiety Inventory: BAI; Beck Depression Inventory – Second Edition: BDI-II; Mini Mental Status Examination: MMSE.

## Competing interests

The authors declare that they have no competing interests.

## Authors' contributions

Conception and design of the Questionnaire: SP, HK, AS, BL. Acquisition of data: SP, HK, AS, CS, BKS, DN, AG, BL. Analysis and interpretation of data: SP, HK, AS, CS, BKS, DN, AG, BL. Drafting the manuscript: SP, HK. Revising it critically for important intellectual content: SP, HK, AS, CS, BKS, DN, AG, BL.

## Pre-publication history

The pre-publication history for this paper can be accessed here:



## Supplementary Material

Additional file 1 The University of Miami Parkinson's Disease Hallucinations Questionnaire (UM-PDHQ) Click here for file
