# Supplementary material for: A questionnaire-based (UM-PDHQ) study of hallucinations in Parkinson's disease
Source: BMC Neurol. 2008 Jun 20;8:21. doi: 10.1186/1471-2377-8-21 (PMC2443167; doi:10.1186/1471-2377-8-21)
Supplement: Additional file 1 — The University of Miami Parkinson's Disease Hallucinations Questionnaire (UM-PDHQ) [file 1471-2377-8-21-S1.pdf]

The University of Miami Parkinson's disease Hallucinations Questionnaire (UM-PDHQ)

Patient identifier:<br>DATE:

	Question	A:Features/Comments	B:Score (circle appropriate)
Severity of hallucinations	1. Do you experience hallucinations? (Have you noticed anything unusual about your vision? Have you had any unusual visual experiences? Or ever see/hear/feel/smell/taste things that are not really there or that other people do not see?)	Type: (mark appropriate)<br>1. Visual<br>2. Auditory<br>3. Somatic/Cutaneous<br>4. Gustatory<br>5. Olfactory<br>(assess each separately)	0. No hallucinations (skip to Annex)<br><br>1. One type only<br>2. Combination<br><br>**C:** Not within the past month, but it has happened in the past
	2. How often do you experience hallucinations?		0 = Only a few times<br>1 = Occasionally (less than once a week, but continuously)<br>2 = Often (about once per week)<br>3 = Frequently (several times per week but < than once per day)<br>4 = Very frequently (≥once per day)
	3. On average, how long do the experiences last?		0 = Short Duration (< 1sec)<br>1 = Medium Duration (< 10secs)<br>2 = Prolonged Duration (> 10secs)
	4. Do you think what you are seeing/experiencing is real?		0 = Not real<br>1 = Sometimes real<br>2 = Always real
	5. How many types of images/sensations do you experience?		1 = One<br>2 = Few (2 or 3)<br>3 = Several (more than 3)
	6. How severe/emotionally distressing do you find these images/sensations or visions?		0 = No effect/Friendly<br>1 = Mildly – produce little distress<br>2 = Moderately – produce distress and are disturbing and disruptive<br>3 = Severely – very disturbing (medications may be required)
	Total Score (min = 0; max = 14)		

Comments:

	Please circle the appropriate answer and provide information	
Quality of hallucinations	7. Have you been diagnosed with any eye disease? (i.e. near or far sight problems, double vision, cataract, glaucoma, retinitis, retinal detachment, diabetic or hypertensive eye disease)	Yes (please describe)<br>No
	8. What are your current medications?	*Complete medication data on page 4.*
	9. Was there a recent change in your treatment? Please describe.	Yes (please describe)<br>No
	10. Was this change related to the appearance or change in the characteristics of hallucinations?	Yes<br>No<br>I cannot tell<br>N/A
	11. Do you experience hallucinations while "on" or "off"?	On<br>Off<br>Anytime-not related to on-offs
	12. What do you normally see/feel/hear/smell/taste?<br>If not visual describe here:<br>Voices, Music, tastes, smells, skin related:	Not formed/cannot describe<br>Whole Faces<br>Fragmented faces ☐ Familiar<br>Whole people ☐ Unfamiliar<br>Animals<br>Insects/reptiles<br>Objects
	13. Is there anything you can do to make the images/sensations disappear?	Yes<br>No
	14. At what time of the day or under which lighting conditions do you experience hallucinations	A. Specific time<br>During the day/Bright<br>During the night/Dark<br>Dim<br>B. Anytime
	15. Do the images ever make any sound or noise (for visual hallucinations)?	Yes<br>No<br>N/A (for non-visual hallucinations)
	16. Do images move (for visual hallucinations)?	Yes<br>No<br>N/A (for non-visual hallucinations)
	17. Are the images normal size?	Yes<br>No, smaller than normal<br>No, larger than normal<br>N/A (for non-visual hallucinations)
	18. Are the images transparent or solid?	Transparent<br>Solid<br>N/A (for non-visual hallucinations)
	19. Are the images colored?	Yes<br>No, (black and white)<br>N/A (for non-visual hallucinations)
	20. Is the onset of hallucinations gradual or sudden?	Gradual (appear-disappear slowly)<br>Sudden (appear-disappear suddenly)<br>I cannot tell
